# Blood Stain Classification with Hyperspectral Imaging and Deep Neural Networks

**DOI:** 10.3390/s20226666

**Published:** 2020-11-21

**Authors:** Kamil Książek, Michał Romaszewski, Przemysław Głomb, Bartosz Grabowski, Michał Cholewa

**Affiliations:** Institute of Theoretical and Applied Informatics, Polish Academy of Sciences, 44-100 Gliwice, Poland; mromaszewski@iitis.pl (M.R.); przemg@iitis.pl (P.G.); bgrabowski@iitis.pl (B.G.); mcholewa@iitis.pl (M.C.)

**Keywords:** hyperspectral classification, deep learning, deep neural networks, forensic science, convolutional neural networks, recurrent neural network

## Abstract

In recent years, growing interest in deep learning neural networks has raised a question on how they can be used for effective processing of high-dimensional datasets produced by hyperspectral imaging (HSI). HSI, traditionally viewed as being within the scope of remote sensing, is used in non-invasive substance classification. One of the areas of potential application is forensic science, where substance classification on the scenes is important. An example problem from that area—blood stain classification—is a case study for the evaluation of methods that process hyperspectral data. To investigate the deep learning classification performance for this problem we have performed experiments on a dataset which has not been previously tested using this kind of model. This dataset consists of several images with blood and blood-like substances like ketchup, tomato concentrate, artificial blood, etc. To test both the classic approach to hyperspectral classification and a more realistic application-oriented scenario, we have prepared two different sets of experiments. In the first one, Hyperspectral Transductive Classification (HTC), both a training and a test set come from the same image. In the second one, Hyperspectral Inductive Classification (HIC), a test set is derived from a different image, which is more challenging for classifiers but more useful from the point of view of forensic investigators. We conducted the study using several architectures like 1D, 2D and 3D convolutional neural networks (CNN), a recurrent neural network (RNN) and a multilayer perceptron (MLP). The performance of the models was compared with baseline results of Support Vector Machine (SVM). We have also presented a model evaluation method based on t-SNE and confusion matrix analysis that allows us to detect and eliminate some cases of model undertraining. Our results show that in the transductive case, all models, including the MLP and the SVM, have comparative performance, with no clear advantage of deep learning models. The Overall Accuracy range across all models is 98–100% for the easier image set, and 74–94% for the more difficult one. However, in a more challenging inductive case, selected deep learning architectures offer a significant advantage; their best Overall Accuracy is in the range of 57–71%, improving the baseline set by the non-deep models by up to 9 percentage points. We have presented a detailed analysis of results and a discussion, including a summary of conclusions for each tested architecture. An analysis of per-class errors shows that the score for each class is highly model-dependent. Considering this and the fact that the best performing models come from two different architecture families (3D CNN and RNN), our results suggest that tailoring the deep neural network architecture to hyperspectral data is still an open problem.

## 1. Introduction

Hyperspectral imaging (HSI) records the spectral data in the visible (VIS) and near- or short wave infrared (NIR/SWIR) ranges. This allows to observe wavelength-specific interaction of light photons with molecules in the observed scene. Those interactions, identifiable through analysis of the pixels’ spectra, can be used to infer the presence or absence of certain materials in the imaged scene. When complemented with the context of the imaging process, this allows for non-destructive assessment of a scene, e.g., a detection of organic compounds [1], classification of crops [2] or food decay verification [3].

In this paper, we focus on the problem of hyperspectral classification. In previous years, a number of methods have been proposed for solving the general hyperspectral classification problem [4]. Hyperspectral classification methods can be divided into spectral only classifiers, e.g., Support Vector Machine (SVM) [5] applied to single pixel information, or spatial–spectral [6] approaches which supplement spectra information with pixel position in the image. Since typically the number of training examples is low in HSI, semi-supervised methods [7], which use both labelled and unlabelled data, are sometimes used to improve the accuracy. Recently, deep learning neural networks [8] have been applied to hyperspectral data classification, following their success in solving general computer vision tasks [9]. Various network architectures were used for building deep hyperspectral classifiers [8], e.g., spectral only convolutions [10], spatial-spectral convolutions [11], autoencoders [12], semi-supervised approaches [13] and recurrent networks [14].

In the authors’ opinion, a particularly interesting case study of hyperspectral classification may be defined in relation to the problem of blood stain identification. Detecting dried patches of blood in a physical scene is an important problem in forensic science [15], while analysis of blood spectral components within the skin can be used for wound severity estimation [16]. The hemoglobin derivatives present in the blood, such as oxyhemoglobin (oxyHb) and methemoglobin (metHB), have characteristics peaks in VIS range spectra, referred to as β (≈542 nm) and α (≈576 nm), which can be used to detect blood or distinguish it from other substances [17]. Moreover, time-inducted decay following the exposure to the elements leads to changes in spectra, which can be used to assess the age of the stains [18].

To explore this problem, we perform two kinds of experiments. The first is a typical scenario of a single image hyperspectral classification, similar to experiments done on Indian Pines and University of Pavia datasets e.g., in [19] or [4]. In this scenario the classifier is trained on a subset of pixels with known labels, sampled from the image itself, and is expected to assign a label to the remaining pixels. We can find similarities to the concept of *transductive learning* proposed by Vapnik [20]. This is because the set of observations is known and limited to pixels in the image and the task is focused on assigning a label to unlabelled observations in this set. Therefore we call this a *Hyperspectral Transductive Classification* (HTC) scenario. In HTC the training set is a good representation of data, therefore we can usually expect high accuracy, even for small training sets [7].

**Figure 1:**
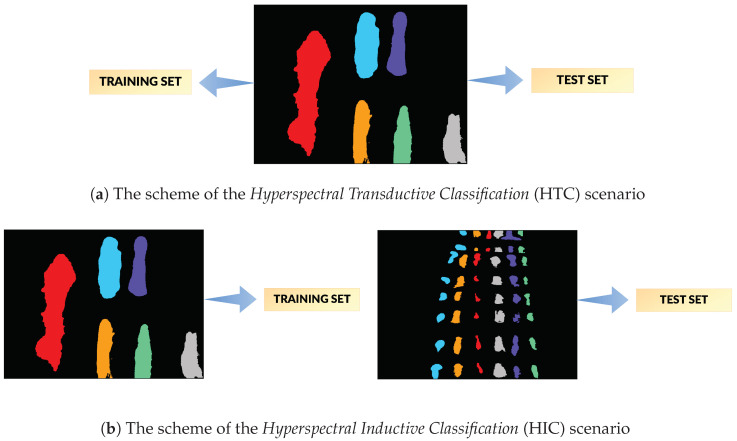
The visualization of the classification scenarios tested in our research. The first row presents the basic scenario in which a training and a test set come from the same image (HTC) while the second row shows the case when a test set is chosen from the another image than a training set (HIC). The HIC scenario is more useful from the point of view of forensic science.

In the second experiment, the classifier is trained on a subset of pixels from one image and tested on an image of a different scene, containing the same materials. This experiment simulates a forensic application, i.e., the classifier is prepared in laboratory conditions and used in the process of a forensic scene analysis. The task is more challenging, compared to the HTC scenario, due to significant differences between training and test images. These differences result e.g., from variations in lighting, the fact that class spectra are mixed with spectra of different, diverse backgrounds [21] and from different acquisition times for images which results in age-inducted changes in class spectra. Because the task is focused on constructing of an accurate classifier function and on a generalization, by analogy to *inductive learning*, we call this scenario a *Hyperspectral Inductive Classification* (HIC). The schema of the HTC and HIC scenarios are presented in Figure 1.

We are particularly interested in comparing, in these two scenarios, the accuracy of classifiers that belong to one of the leading groups of machine learning methods, namely deep neural networks (DNNs). DNNs are known to be able to extract complex features, and they perform very well in the HTC scenario [22]. However, a question arises: how do they perform for images with different spatial and spectral structure and in a more challenging scenario? We intend to answer this question by applying a diverse set of deep learning architectures to blood stain classification problem in the HTC and HIC scenarios. For this task we use a dataset [23] which contains several hyperspectral images of blood and blood-like substances (such as ketchup, artificial blood, tomato concentrate, poster and acrylic paint). These substances are visually similar and their distinction based on traditional photos is difficult. The images in the dataset simulate different acquisition scenarios (laboratory, crime scene, blood splatter), and were captured at different time intervals. Annotation of classes presented in images is provided by their authors [23].

For our experiments, we selected different state-of-the-art architectures of neural networks (convolutional, recurrent and multilayer perceptron) designed for HSI analysis. Their efficiency was compared with multilayer perceptron and Support Vector Machine classifiers.

Our main contributions are:

1. We perform a study of blood stain classification from hyperspectral images with deep neural networks. To the best of the authors’ knowledge, this study is the first of its kind.
2. Through the presented case study, we investigate the performance of deep neural networks on a real-life hyperspectral dataset that complements the typical tests done on remote sensing images e.g., Indian Pines or University of Pavia images. While the remote sensing transductive scenario is popular, we argue that the proposed dataset is, in terms of scene contents and acquisition conditions (camera distance, lighting, image preprocessing), close to many practical applications (e.g., food and materials inspection, forensic detection, medical imaging). While individual papers introducing DNN architectures (e.g., [24] or [11]) present comparisons to a selection of reference algorithms, the authors are aware of only two studies [8,19] that present a broad comparison and discussion of methods, both focus on remote sensing data. Our study extends that work by discussing the performance of networks in a different, ‘local’ sensing.
3. We compare the performance of transductive and inductive hyperspectral classification. The inductive classification scenario is much less investigated, while at the same time it is both more difficult and relevant for practical application.

### Related Work

Sensitivity to hemoglobin makes hyperspectral imaging a promising tool for medical diagnosis [16] and non-invasive blood detection [25]. For this reason, blood detection has been extensively studied, e.g., in [26] blood was spilled on various multicoloured materials along with other substances like red wine or lipstick. Authors pointed out that blood is harder to detect on darker materials. A similar study was described in [27] where HSI is applied for detection of bloody fingerprints. The process of hemoglobin degradation can be observed through changes in reflectance spectra [28], which can be used to assess the age of blood. This problem was studied in [29]; in their experiments, the authors used various clustering algorithms on samples aged up to 200 days. They observed that blood age estimation can be a challenging task without the knowledge about the acquisition environment.

Another actively studied research area is a hyperspectral data classification [4]. Different machine learning methods like SVM [30], random forests [31] or neural networks such as Extreme Learning Machines (ELM) [32] were used. Recently, deep neural networks have become popular, following their successful application for classic image classification [33,34] and object detection [35]. Examples of architectures employed for HSI classification include one [10], two [36] or three-dimensional [22] convolutional models for more efficient use of spatial and spectral information [37].

Deep learning architectures have been actively developed over the recent years. An up-to-date summary of the latest architectures used for hyperspectral classification, along with a discussion of results for common datasets can be found in [8,19]. Another wide comparison of neural network architectures for well-known HSI datasets is presented in [38]. What is particularly interesting in the latter study is that the authors performed experiments with relatively small training sets and discussed the impact of data augmentation, transfer learning and residual learning on classification accuracy. The problem of learning from a limited training set is important in HSI classification, as training labels are often difficult to obtain [7]. Typically, deep learning requires a large number of labelled examples; therefore, the emergence of architectures designed to limit this requirement, such as e.g., [39] is promising. Another novel, interesting approach is the use of hybrid dilated residual networks, which is presented in [40]. Sometimes, deep learning is combined with classic machine learning algorithms. For example, in [41], the authors presented a hybrid classification method which combines deep learning with SVM. Another example is the method based on deep metric learning presented in [42].

In the inductive classification scenario in our experiments, features of training and test images are different. It is a demanding scenario for a classifier, similar to the problem studied as a covariate shift in [43]. To alleviate the impact of such differences in data, authors of [44] propose the density ratio estimation. In the field of deep learning, such a problem is related to transfer learning and domain adaptation e.g., in [45] authors train their networks on the source dataset and try to find the most efficient way of fine tuning the network to the target. It turns out that saving some layers from the pretrained network while retraining the others with new data can increase the classification accuracy. On the other hand, authors in [46] present the transductive algorithm applied in the feedforward neural network. Its main purpose is to improve the global classification accuracy on the dataset using a subset of the input vectors, called prototypes. This approach is compared to the inductive case.

## 2. Methods

In our experiments, we tested six state-of-the-art deep learning network architectures for hyperspectral imagery from DeepHyperX library [19]: a multilayer perceptron, four convolutional neural networks (CNNs) and one recurrent neural network (RNN). Results were also compared with a Support Vector Machine (SVM) classifier as a standard reference algorithm that achieves competitive results in hyperspectral classification [4]. In this section we describe the chosen architectures and their parameters. All networks hyperparameters, such as the number of neurons, kernel sizes, activation functions, number of epochs, batch size, etc., were taken from their implementation in DeepHyperX [19] (unless explicitly stated otherwise like in the case of 1D CNN [10] architecture). The authors in [19] declare that their implementation is as similar as possible to the approaches of individual authors. We rely on optimization of hyperparameters done by the authors of referenced papers. Our objective was to keep our results comparable with [19].

### 2.1. Multilayer Perceptron

**Figure 2:**
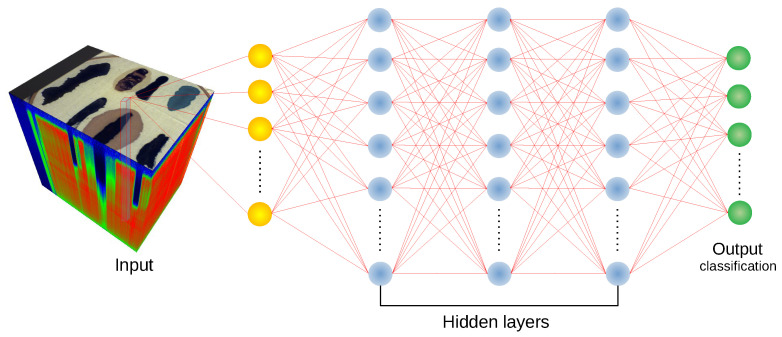
Schematic of the MLP architecture [19] used in experiments.

Despite its simplicity, a multilayer perceptron [47] achieves high accuracy in hyperspectral classification tasks [4] and is often used as a reference for more complicated architectures [19]. The implementation used in our experiments consists of three hidden layers (2048, 4096 and 2048 neurons), with cross-entropy loss function [19]. The scheme of the MLP architecture is presented in Figure 2.

### 2.2. Deep Recurrent Neural Network

**Figure 3:**
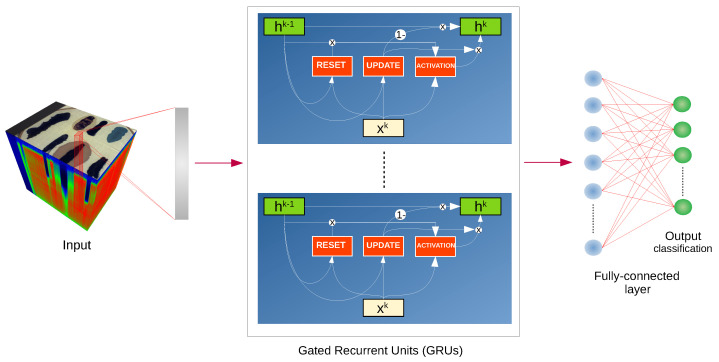
Schematic of the RNN architecture [14] used in experiments, we denote the value of k-th spectral band as $x^{k}$ and the hidden state of the previous and the current step as $h^{k-1}$ and $h^{k}$, respectively.

In [14], the authors propose a deep recurrent neural network (RNN) that uses gated recurrent units (GRU). The RNN architecture includes loops in its design, which allows it to process sequential data, i.e., data where the output for a given example depends on the previous examples processed by the network. GRU unit was designed to work effectively with long-term sequences but in contrast to the long short-term memory (LSTM) unit it also has fewer parameters. The authors treat hyperspectral pixels as sequences to train the RNN. To produce bounded and sparse output authors propose new activation function *PRetanh* given by the following equation:

(1) $$f\left(a_{i}\right)=\left\{\begin{array}{cc} \tanh(a_{i}), & a_{i}>0,\\ \lambda_{i}\tanh\left(a_{i}\right), & a_{i}\leq 0, \end{array}\right.$$

where $a_{i}$ is the input of the activation *f* for the *i*-th channel and $\lambda_{i}\in \left[0,1\right]$ is a learnable parameter which can differ for different channels. The network diagram with sketch of GRU layer is presented in Figure 3.

The GRU layer has a size of 64 units with sigmoid gate activation and *PRetanh* activation function for hidden representations. To address the problem of small and unbalanced training set, authors utilize dropout with probability of 0.5 on the output of recurrent layer, as well as a dropout of 0.2 on the weight matrices of the proposed model. Moreover, the batch normalization is used. In the output layer the softmax activation function is utilized to provide class probabilities. The cross-entropy loss function is used to train the network.

### 2.3. Convolutional Neural Networks

Convolutional neural networks [47] (CNN) use convolutional layers which create feature maps from input data followed by pooling layers that reduce dimensionality of feature maps. When used to process hyperspectral images, depending on the type of the network, the convolutions can be defined as:

- one-dimensional, taking into account only spectral vectors and ignoring spatial relationships between pixels;
- two- and three-dimensional, exploiting local neighbourhoods of hyperspectral pixels and spatial-spectral relationships.

This flexibility in defining the convolution allows to customize the network for different types of hyperspectral data. In most of the architectures, convolutional layers are followed by fully connected layers that classify examples using extracted features. CNNs may also use dropout regularization that randomly removes neurons with a predefined probability to avoid overfitting.

#### 2.3.1. 1D Convolutional Neural Network

**Figure 4:**
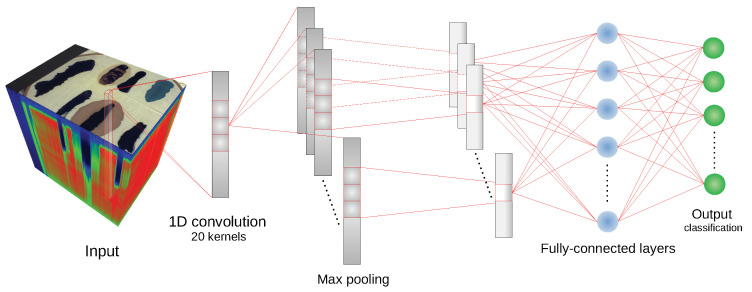
Schematic of the 1D CNN architecture [10] used in experiments.

One of the proposed CNNs applied for the presented HSI classification problem is the architecture based on [10]. It consists of one 1D convolutional layer, one 1D max pooling layer and two fully connected layers. Its advantage is the simplicity of the model. The convolutional layer is composed of 20 kernels of size $c=\left\lceil \frac{n_{B}}{9}\right\rceil$, where $n_{B}$ is the number of hyperspectral bands. In the next step, data is processed by the max pooling layer with the kernel size $m=\left\lceil \frac{c}{5}\right\rceil$. During these two stages padding and dilation are equal to 0 and 1, respectively. For the convolutional layer the stride is set to 1 while for the pooling layer it equals *m*. Then, the extracted features are inserted into the fully connected layer of 100 neurons. Finally, the last layer is responsible for the classification (the number of neurons equals the number of classes). As a loss function the cross-entropy is chosen. The illustration of the described network is shown in Figure 4.

#### 2.3.2. 2D Convolutional Neural Network

We use the 2D architecture proposed in [24]. First, data is processed with a 2D convolutional layer with kernels of different sizes, which the authors called a multi-scale filter bank. The authors emphasized the similarity to the Inception module [48]. In the implementation from [19], two filters were applied: 3×3 and 1×1 and results of filtering are combined. Then, data is processed by multiple 2D convolutional layers. Each layer except the last one (classification layer) has 128 filters. After the multi-scale filter bank, as well as the 2nd, 3rd, 5th, 7th and 8th convolutional layers, the ReLU(·) activation function was used.

**Figure 5:**
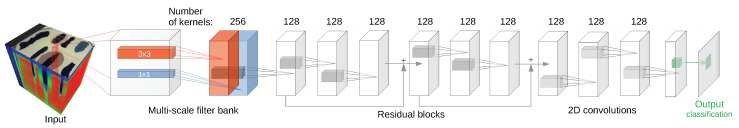
Schematic of the 2D CNN architecture [24] used in experiments.

In addition, two residual connections are used, which improve the gradient flow through the network and local response normalization (LRN) is used after the first two convolutional layers, which normalizes activations of neighbouring filters. Dropout was applied after 7th and 8th layers. Similarly, as in the previous models, the cross-entropy is used for the training. The schema of this network is presented in Figure 5.

#### 2.3.3. 3D Convolutional Neural Network

**Figure 6:**
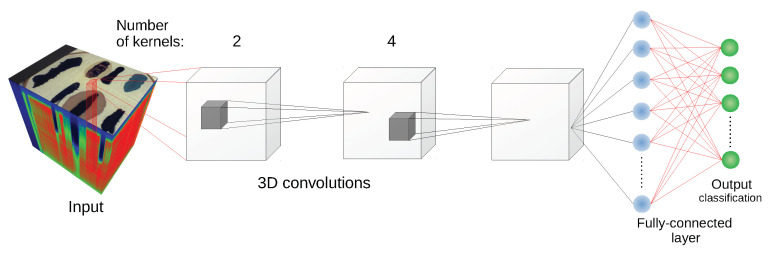
Schematic of the 3D CNN architecture [11] used in experiments.

We used two architectures of this type in our experiments. The first one is the architecture presented in [11], consisting of two 3D convolutional layers and one fully connected layer (without pooling layers), as can be seen in Figure 6 where the schema of the network is presented. Kernel sizes (width, height, depth) were set as (3,3,7) and (3,3,3) respectively, and padding dimensions are (1,0,0) in both layers. The second convolutional layer has twice as many kernels as the first one. Furthermore, after each convolutional layer, the ReLU(·) activation function was applied.

**Figure 7:**
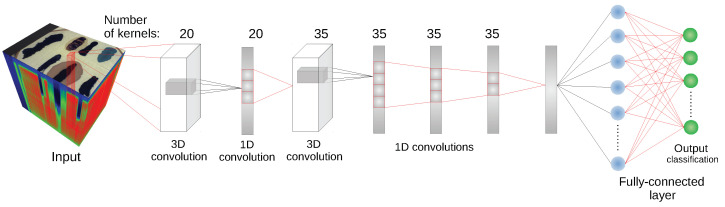
Schematic of the 3D CNN architecture [49] used in experiments.

The second 3D CNN we used is based on [49] and is presented in Figure 7. Apart from 3D convolutional layers, this architecture uses 1D convolutions. The pipeline of this model consists of two 3D convolutional layers with kernel size (3,3,3) and stride equal to 1 intertwined with two 1D convolutional layers with kernel size (1,1,3) and stride set to 2. Furthermore, the authors use two consecutive 1D convolutional layers with kernel sizes (3,1,1) and (2,1,1), respectively, and strides equal to 1 and 2, respectively. Finally, one fully connected layer with softmax function was used to create class probabilities. For the two convolutional layers, the number of neurons was set to 20 while for the remaining convolutional layers it was 35. The ReLU(·) activation function was used for all layers except for the first two 1D convolutions. The two 3D CNNs are trained by using the cross-entropy loss function.

#### 2.3.4. Performance Measures

In order to compare the presented methods, we used the following metrics: Overall Accuracy (OA), Average Accuracy (AA), Cohen’s kappa coefficient (κ) and a method based on t-SNE projection of confusion matrices. OA can be described as a ratio of correctly classified pixels to the total number of pixels. AA is computed as an arithmetic mean over all classes from ratios of properly classified pixels belonging to a given class to the total number of pixels from this class. This metric allows for the detection of discrepancy in the prediction results of individual classes. κ coefficient [50] compares the agreement of two discrete probability distributions and checks their difference of judgement, according to the equation:

(2) $$\kappa =\frac{p_{o}-p_{c}}{1-p_{c}},$$

where $p_{o}$ denotes the relative observed agreement that samples belong to the given class (the accuracy) while $p_{c}$ indicates the probability that the samples match randomly. In our case, the ground truth is compared with the predictions of classifiers.

In addition, a method based on t-SNE projection is used for outlier identification in the results and is described in detail in Section 3.3.2.

## 3. Results

This Section presents the details of performed classification experiments: the dataset, the procedure, evaluation method, results and discussion.

### 3.1. Evaluation Dataset

Hyperspectral images used in our experiments come from the dataset described in [23], which is publicly available under an open licence (the dataset with snippet code for data loading: https://zenodo.org/record/3984905). The dataset consists of annotated hyperspectral images of blood and six other visually similar substances: *artificial blood*, *tomato concentrate*, *ketchup*, *beetroot juice*, *poster* and *acrylic paint*. Images in the dataset were recorded on the course of several days, to capture changes in spectra related to the process of the time-related substance decay. On every image, hyperspectral pixels where classes are visible were annotated by authors, therefore they can be treated as labelled examples i.e., pairs $(x_{i},l_{i})\in X$ from a set of labelled examples X, where vectors $x_{i}\in R^{d}$ are hyperspectral pixels, d∈N denotes the number of bands and $l_{i}\in N$ are class labels.

The majority of images in the dataset were captured using an SOC710 hyperspectral camera operating in spectral range 377–1046 nm with 128 bands. Following suggestions in [23] we removed bands [0–4], [48–50] and [122–128], leaving 113 bands. From six image types in the dataset we decided to use two that are most useful for our experimental setting. First, the *frame* scenes, denoted as *F* in [23], present class examples on a uniform, white fabric background; this image has the advantage of distinct separation and clear spectral characteristic of the substances which makes it a good benchmark dataset for tested models. The other type of images that was used is the *comparison scenes*, denoted as *E*. These present the same substances on diverse backgrounds consisting of multiple materials and fabrics types. This means that substance spectra are mixed with the background spectra, which provides a more challenging classification setting. Since one of the classes, the *beetroot juice* was not present in all images, it was removed in our experiments, leaving six classes.

**Figure 8:**
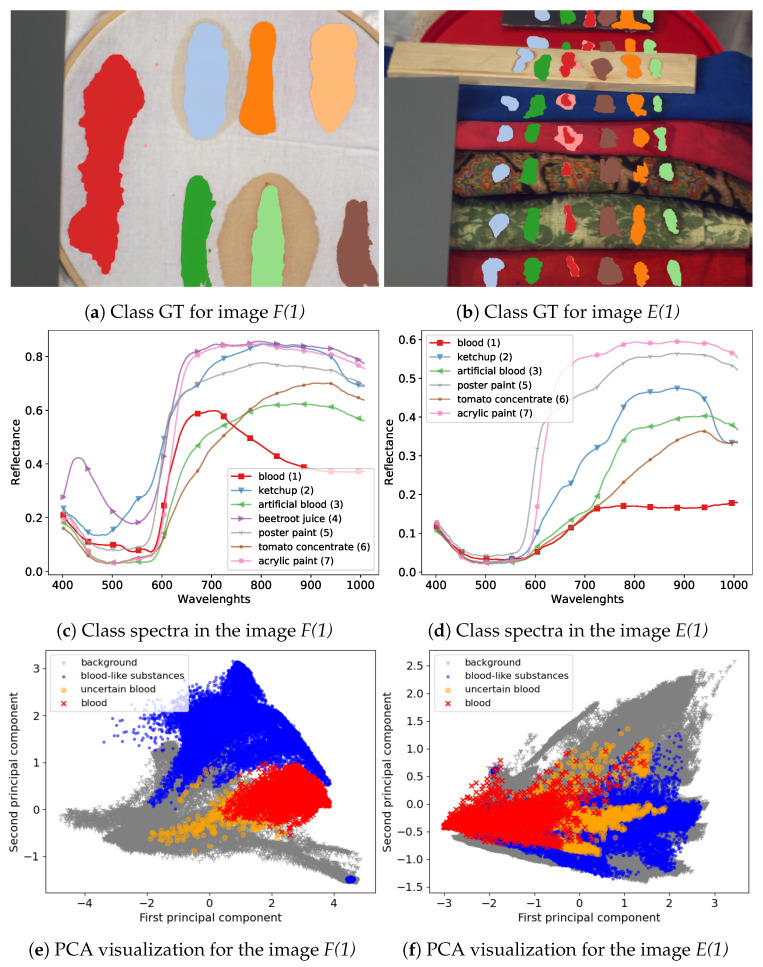
Dataset visualization. Upper panels present classes as a coloured ground truth on RGB images created from hyperspectral cubes. Middle panels present mean class spectra. Bottom panels present PCA projection of data on first two principal components. The images come from [23].

Additionally, we used images captured in different days to take into account changes in the blood spectrum over time. For both scene types we used images from days {1,7,21} (6 images in total), denoted *F(1), F(7), F(21)* and *E(1), E(7), E(21)*, respectively. For scenes *F* we additionally used an image denoted *F(1a)*, taken sever hours later than the image *F(1)* and two images obtained in the second day, denoted *F(2)* and *F(2k)*. The image *F(2k)* was captured with a different hyperspectral camera. Spectra in this image were linearly interpolated to match bands from the SOC710 camera. A more detailed description of the dataset can be found in [23]. Visualization of example images from the dataset and a comparison of their spectra are presented in Figure 8.

### 3.2. Experimental Procedure

We performed two types of classification experiments (see Section 1 for motivation and discussion):

1. *Hyperspectral Transductive Classification* (HTC) scenario treats every image in the dataset separately. In every experiment, the set of labelled pixels in the image X is divided between the training and the test set, i.e., $X_{\mathrm{train}}\subset X,X_{\mathrm{test}}\subset X,X_{\mathrm{train}}\cap X_{\mathrm{test}}=\emptyset$.
2. *Hyperspectral Inductive Classification* (HIC) uses a pair of images: a set of labelled pixels from one image is used to train $X_{\mathrm{train}}\subset X_{1}$ the classifier which is later tested on full set of labelled pixels $X_{\mathrm{test}}=X_{2}$ from the second image.

Based on the discussion in [23], pairs of images for training and testing the classifier were chosen in the HIC experiment: a *F/frame* is used for training, as it simulates the lab acquired reference data, while a *E/comparison scene* image is used as test, as it simulates a real-life crime scene. The complexity of HIC is enhanced by the fact that not every *F* image has its *E* counterpart acquired at the same time. Among pairs are ones with corresponding acquisition times (e.g., *F(7)→E(7)*), acquisition times differing by hours (e.g., *F(1a)→E(1)*) or days (e.g., *F(2)→E(7)*). Those differences are introduced on purpose, to allow examination of the effect of time-related change of spectral shape on the classification performance. For similar reasons, we have included two pairs *F(2)↔F(2k)*; while they share a similar scene, they were acquired with different cameras and thus allow to investigate the effect of the equipment change.

**Figure 9:**
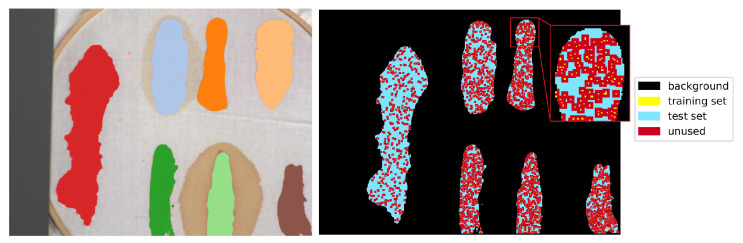
An example of the train and test set selection for the *F(1)* image. The left panel presents a RGB visualization with superimposed ground truth class labels. The right panel presents selected training ($5\%\cdot n_{s}$, where $n_{s}$ is the number of pixels of the least numerous class) and test examples. Pixels within 2-pixels neighbourhood of every training point are not used for testing to avoid non-empty intersection between training and test sets for networks that process patches of pixels.

Since some of the architectures considered (e.g., 2D CNN [24] or 3D CNN [11]) use a block of pixels as an input, there was a need to create a uniform training/testing picture split for every image. The objective was to avoid patches from the training set having non-empty intersections with patches from the test set. For each class, a set of $n_{train}=5\%\cdot n_{s}$ samples, where $n_{s}$ is the number of pixels of the least numerous class, was randomly, uniformly selected as training pixels. Each class was thus represented by the same number of samples. The 2-pixel neighbourhood of the training set—a maximal size of the neighbourhood used by tested architectures—was marked as unusable, along with the margin at the image borders of the same dimension. All of the remaining labelled pixels formed a test set. An example illustration of the effect of this procedure is presented in Figure 9. For every image $n_{sets}=10$ different sets of randomized training pixels were prepared; the same collection of training sets was used for each network, ensuring comparability of results between individual architectures. The results for each scenario of network type/image configuration were averaged over 10 runs with the above specified fixed training datasets. Given 17 image scenarios, seven methods and ten runs for each image/method configuration, the total number of runs was $n_{runs}=1190$.

We have used the implementation of network architectures provided with DeepHyperX library [19]. The models were initialized with the default values of hyperparameters as distributed with the library. Initial trial runs were performed to verify the general performance and assess the stability and suitability of the hyperparameter choice. In all but one case the default parameters were used for the main experiment. For the one case—1D CNN [10] architecture—a minor adjustment was made: number of epochs was increased to $n_{e}=400$ and batch size was decreased to $n_{b}=50$. While there exists a possibility that extensive hyperparameter tuning would improve performance of the models, it would also make it more difficult to compare our results with [19] and assess generalization capability of each network. By applying the architectures to our data with similar hyperparameters as in [19], we broaden the perspective while at the same time testing the robustness of the default hyperparameter choice. Additionally, hyperparameter optimisation requires a high degree of intimacy with a given architecture; this poses a risk to produce more effective optimisations for some models than the others, thus introducing a bias in the results. Too much optimisation may overtrain a model to a given dataset, which would introduce additional bias, and would go against our objective of ‘stress testing’ the selected architectures.

For the reasons discussed in the previous paragraph, we have not used any preprocessing that would significantly transform the spectra. The only preprocessing performed, as suggested in [23], was spectra normalisation: for each image, every hyperspectral pixel vector was divided by its median. This procedure is intended to compensate for non-uniform lightning exposure.

As a reference for the tested deep learning architectures, we tested the SVM classifier [51]. For its construction, we have used the Radial Basis Function (RBF) kernel and parameters chosen through internal cross-validation from a range of $C\in \langle 10^{-3},10^{3}\rangle$ and $\gamma \in \langle s\times 10^{-3},s\times 10^{3}\rangle$, where $s=\frac{1}{n_{f}\times v}$ with $n_{f}$ is the number of bands and *v* is the variance of data.

### 3.3. Evaluation Procedure

#### 3.3.1. Implementation

Experiments were performed using Python 3.7.6 (64-bit). Except for the DeepHyperX [19], the following libraries were used: PyTorch 1.5.0 [52], scikit-learn 0.22.1 [53], CUDA Toolkit 10.2.89, numpy 1.18.1 [54], matplotlib 3.1.2 [55], spectral 0.21, scipy 1.4.1, torchsummary 1.5.1 and conda 4.8.2.

The source code for replication of experiments can be found on the github repository (Source code location: https://github.com/iitis/DeepHyperBlood).

#### 3.3.2. Evaluation Metrics

To compare tested methods we used three measures of classification performance: Overall Accuracy (OA), Average Accuracy (AA) and Cohen’s kappa coefficient (κ), which are often used to evaluate the result of hyperspectral classifiers [4].

**Figure 10:**
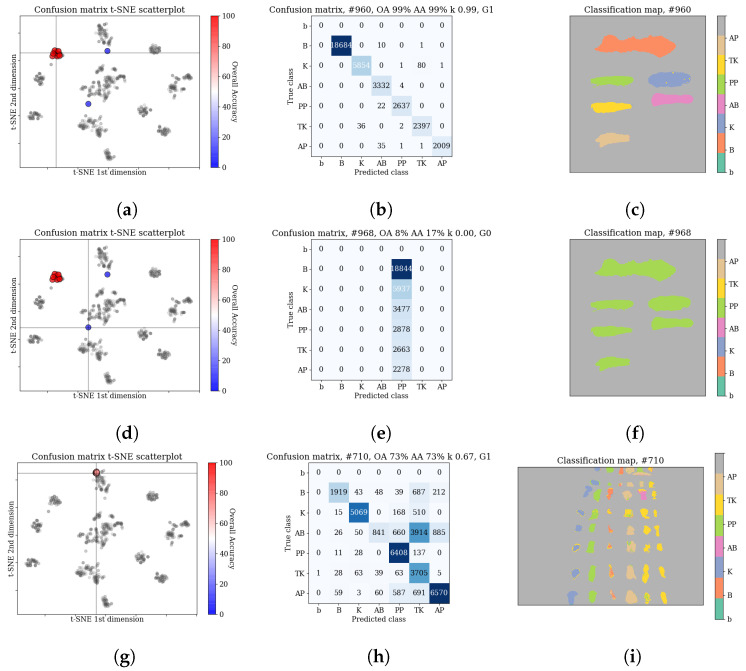
Example steps of the qualitative inspection of the experimental results (see Section 3.3.2). The rows present three separate inspected cases. The columns contain the inspected data: a scatterplot of t-SNE projected confusion matrices, the case confusion matrix and a classification map. On the scatterplot the grey points represent classification results for all images, while the marked points are connected with the presented case. Scores of the relevant scenario (network/images) were emphasized. The first two rows present the scenario of the 3D CNN [11] network on *F(2k)* image; the result is relatively high and comparable with others results with mean OA of 99.2%±1.7%, it contains two detectable outliers, which results from failure of training. Inclusion of them in the score would drop the mean OA to 80.9%±36.7%, which would hide the real capabilities of this network. Such results were thus excluded. Bottom row: the RNN [14] network on *F(1a)→E(1)*; consistent performance verified by the low dispersion of the confusion matrices and their similar shape for subsequent runs. (**a**) Confusion matrix scatterplot, 3D CNN [11] network, *F(2k)*, (**b**) Confusion matrix for case selected in (**a**), (**c**) Classification map for case selected in (**a**), (**d**) Confusion matrix scatterplot, 3D CNN [11] network, *F(2k)* (same as (**a**)), (**e**) Confusion matrix for case selected in (**d**), (**f**) Classification map for case selected in (**d**), (**g**) Confusion matrix scatterplot, RNN [14] network, *F(1a)→E(1)*, (**h**) Confusion matrix for case selected in (**g**), (**i**) Classification map for case selected in (**g**).

Additionally, to ensure the quality of the results, we performed a qualitative inspection of all results. For that, we have used the following algorithm. First, confusion matrices for each run were extracted, and projected into a 2D plot using the t-SNE dimensionality reduction algorithm [56]. Each scenario, consisting of a given architecture and image training/test pair, was inspected for dispersion and presence of outliers in the confusion matrix set. The usage of t-SNE projection allowed for enhanced capability of examination, compared to analysis of descriptive statistics (i.e., mean, standard deviation or kurtosis). For example, it allowed us for identification of outliers and comparing the error patterns for each experimental scenario against the total results. An example snapshot of this analysis is presented in Figure 10. A similar analysis was also carried out using t-SNE projected classification maps.

### 3.4. Experimental Results

**Figure 11:**
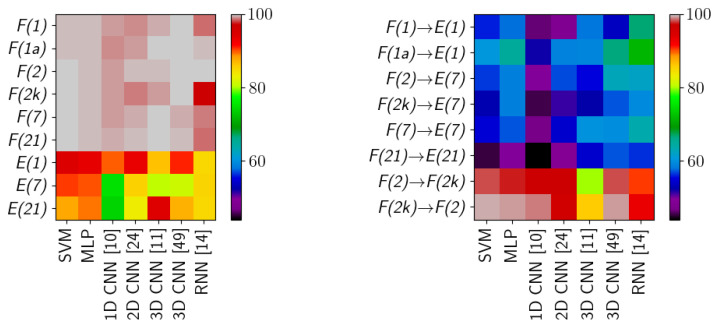
Graphical visualization of results from transductive (HTC, Table 1) and inductive (HIC, Table 2) scenario. The presentation is based on the Overall Accuracy scores.

The qualitative analysis of the results, discussed in Section 3.3.2, identified $n_{fail}=11$ cases of failed network training. In all those cases the predicted labels belonged to a single class (In one of those cases, all but one (13,563 samples) were classified as one class, and a single sample was marked as an another class. For the sake of simplicity, we classify this case as in line with the rest of training failures.). We have removed those cases before computing statistics of the results. The scores—means and standard deviations of OA, AA and kappa—for the transductive classification (HTC experiment) are presented in Table 1, while results for inductive classification (HIC experiment) are presented in Table 2. Additionally, per-class error percentages are presented in Table 3, Table 4, Table 5 and Table 6. A graphical presentation of the results in Table 1 and Table 2 is in Figure 11. The detailed information about running times of applied methods are presented in Appendix A.

The scores for HTC form two groups, clustered around the *F/frame* and *E/comparison scene* type images. For *F/frame* scenes the results are consistently high for all methods, often above 99%. Those images have a simple, uniform white background which does not interfere with individual class spectra. Left alone, those spectra are easily modelled by methods considered in this study. Inspection of an averaged confusion matrix reveals that the most common errors are misclassifications between *ketchup* and *tomato concentrate*, which is not unexpected as they represent similar substances. Qualitative inspection of individual results shows that while the scores are similar, the types of errors for individual methods are distinct, i.e., some models ‘favour’ certain classes (see Table 3). For example, 1D CNN [10] has the highest error rate for *ketchup* (1.7%) and RNN [14] for *tomato concentrate* (5.4%); another example is the range of errors, e.g., *artificial blood* is from 0.2% (3D CNN [49]) to 3.7% (RNN [14]). This suggest that various architectures are able to model different features of the spectra. Quantitative investigation of scatterplots of confusion matrices (see Section 3.3.2) reveals that in a small number of cases, errors are concentrated mostly within a subset of classes, e.g., ‘bloodlust’ when the model has the tendency to label a sample as *blood*, or ‘artistic differences’ when the model tends to misclassify among the *paints* classes. We discuss possible reasons for that in the ‘Discussion’ section.

Results are visibly lower for *E/comparison scene* images, where the decrease in accuracy is about 5–10%. Quantitative inspection reveals that some architectures (SVM, MLP, 1D CNN [10], 2D CNN [24]) have consistent results, i.e., models from different runs have very similar scores and confusion matrices; while other (3D CNN [11,49], RNN [14]) have some share of underperforming models, which have lower accuracy and distinct confusion matrices. The latter effect is responsible for both a lower mean score and the increase in standard deviation. In comparison to a set of scenarios discussed in the previous paragraph, there is a visible change not only in magnitude of errors for individual classes (see Table 4), but also in their order—some classes which had lower error rate now have a higher one. For example, while the *blood* class has a range of errors 9.9–15.6%, the *poster paint* has a range of 1.8–2.5% and *tomato concentrate* 16.4–26.4%. In those images the scene background is composed of objects with complex spectra, which forms spectral mixtures with classes to be recognized, making class spectra more diverse and challenging to model. This phenomenon is known to be present in many hyperspectral images [21], and the enhanced scene complexity translates into lower scores.

The inductive classification (HIC experiment) is a significantly more challenging scenario, where different images are used for training and testing. In some cases, e.g., *F(2k)→E(7)*, the difference can be in: scene composition (*frame* vs *comparison* scene), sample characteristics (two day old samples for training, seven day old for testing) and acquisition equipment (SPECIM vs SOC-710 camera). One of the tested inductive cases is *F(2k)↔F(2)*, where the same frame scene is independently imaged with two different cameras. The mean scores are lower than HTC *F/frame*, but higher than HTC *E/comparison scene*. Some networks have their score lowered by particular classes (e.g., 3D CNN [11] with *tomato concentrate*, 11.2% errors or RNN [14] with *artificial blood*, 38.1%, see Table 5). Some networks (3D CNN [11] in particular) have higher percentage of less performing models, which are responsible for lower scores. The change in acquisition device produces distinct, in relation to other scenarios, patterns in the results. The training on higher quality device (SPECIM, *F(2k)*) produces better scores than the opposite scenario. Both the MLP and the reference SVM classifier achieve high accuracy in this case.

The final case—training on *F/frame* and testing on *E/comparison scene*—is viewed by the authors as a true application-related test of hyperspectral classification. The *F/frame* image simulates laboratory-taken image, which is then used to investigate a sample forensic scene. This adds the variation of classes spectra and lighting on top of complexities already present in the *comparison* scene. The first observation is in that both the reference SVM classifier and the simple MLP architecture are outperformed by more complex models such as RNN [14] or 3D CNN [49]. In this setting the errors of classes *ketchup* and *artificial blood* again dominate (42.7–84.6% and 76.7–85.3% respectively). The *blood* class, detection of which is interesting from an application perspective, has a range of errors 29.4–44.8%. The confusion matrices show large variations and no noticeable patterns.

**Figure 12:**
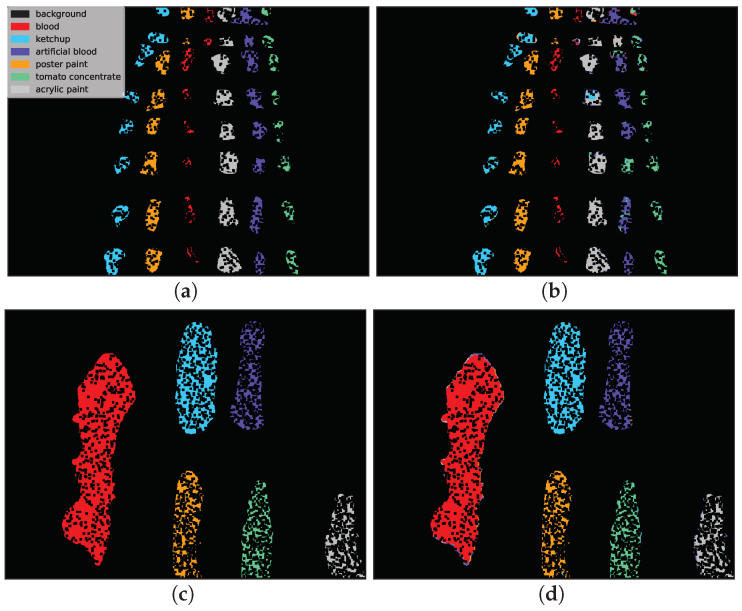
Two selected examples of classification maps for sample runs of the 1D CNN [10] and 2D CNN [24] architectures in the HTC scenario. Most of the classes were correctly labelled in this scenario, which resulted in high classification accuracy. (**a**) Ground truth for *E(1)* image. (**b**) Prediction of 1D CNN [10] network. (**c**) Ground truth for *F(1)* image. (**d**) Prediction of 2D CNN [24] network.

**Figure 13:**
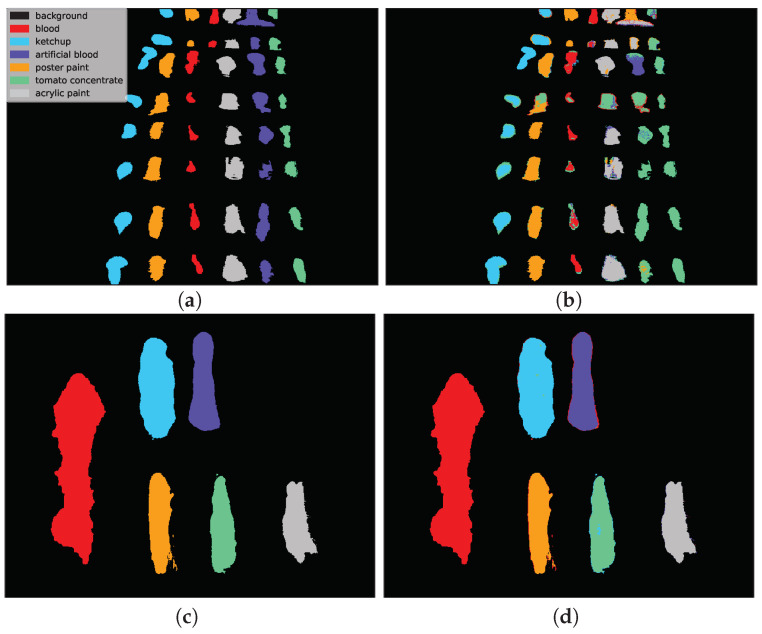
Two selected examples of classification maps for sample runs of the RNN [14] and 3D CNN [49] architectures in the HIC scenario. For the *E(1)* image, where the training spectra originated from a distinct data cube i.e., *F(1)*, some classes (e.g., artificial blood and tomato concentrate) were misclassified. However, for the *F(2k)* image where the training data cube i.e., *F(2)* originated from the same scene, classification errors are minor, despite the fact that both images were acquired with different hyperspectral cameras. (**a**) Ground truth for *E(1)* image. (**b**) Prediction of RNN [14] network. (**c**) Ground truth for *F(2k)* image. (**d**) Prediction of 3D CNN [49] network.

Example classification maps for different architectures in the HTC and HIC scenarios are presented in Figure 12 and Figure 13, respectively. Figure 12 presents example predictions for the HTC scenario: 1D CNN [10] for *E(1)* image and 2D CNN [24] for *F(1)* image. Note that in the HTC scenario only a subset of available pixels could be used as a test set (see Section 3.2) which results in ‘holes’ in classification maps. In the first row we observe misclassifications of *artificial blood* class, where some pixels were incorrectly recognized as *tomato concentrate* and *acrylic paint*. The example in the second row was easier to classify and errors were mainly concentrated on the borders of substances (uncertain areas where spectra are likely to be mixed).

Figure 13 presents the HIC scenario. The first row is related to *F(1) → E(1)* case and presents a sample run for RNN [14] network. One can observe that almost all pixels of *artificial blood* class were incorrectly classified as *tomato concentrate*, *acrylic paint* or *poster paint*. The prediction presented in the second row i.e., 3D CNN [49] network for *F(2) → F(2k)* scenario is highly accurate except for some border pixels.

### 3.5. Discussion

#### 3.5.1. Performance of DNNs in the HTC and HIC Scenarios

As explained in the Section 1, the HTC scenario is a common setting in hyperspectral remote sensing. However, we argue that it limits the possible applications of hyperspectral classifiers. For example, when considering forensic applications, problems such as blood detection [25] and blood age estimation [15] are related to the HIC scenario.

Comparing our results in the HTC in Table 1 and HIC in Table 2, we can see that the average classification accuracy in HTC scenario is much higher. This high performance in HTC confirms the capability of tested ML algorithms to model class spectra and distinguish between classes, despite their visual and physical (e.g., *ketchup* and *tomato concentrate*) similarity. When considering practical application, this could be employed e.g., in the scenario where an expert labels a subset of pixels in the image and uses a classifier as a tool to annotate the remaining pixels. On the other hand, the lower performance in the HIC scenario, shows that the differences between the sources of training and test data are indeed a challenge for classifiers. The extent of these differences may be surprising considering that all images were acquired in laboratory conditions and the substances (classes) are clearly visible and identical in all images. If we look at last two rows of the Table 2, we notice that when imaged areas are similar e.g., *F(2)→F(2k)*, the accuracy is closer to that in the HTC scenario. This indicates that the challenge results come more from the differences in class spectra inducted by various backgrounds or time of application than from differences in lightning, acquisition noise or type of acquisition equipment.

#### 3.5.2. Evaluation of Tested Networks

Some of the observations made as a result of our experiments seem to be common to all tested architectures. In the HTC scenario tested models usually achieved high classification accuracy. In both the HTC and HIC scenarios, some classes, notably *artificial blood* seemed to be more challenging for classifiers while others, e.g., *poster paint* were easier, which seems consistent with detection results in [23]. Interestingly, accuracy for the *blood* class was high in most of the experiments, which may suggest that DNN architectures can utilize its characteristic features. All models were also comparable in terms of their computational performance.

Our observations regarding the tested architectures are as follows:

- RNN [14]—In the reference paper [19] results of this architecture were average among tested methods. In our HIC scenario, this architecture achieved one of the best average accuracies. Interestingly, it performed particularly well for *F(·)→E(·)* scenarios. On the other hand, in simpler scenarios such as e.g., *F(2)↔F(2k)* it was on average less accurate than other models. In some experiments e.g., HTC scenario *F(1)* or *E(1)* its results had a large standard deviation. Analysis of per-class scores (see Table 3, Table 4, Table 5 and Table 6) reveals that the model made significant errors for the *artificial blood* class.
- 3D CNN [49]—Similarly to RNN [14], while not exceptional in [19], in our HIC scenario this architecture achieved one of the highest average accuracies among tested models. In addition, it also scored high in the *F(2)↔F(2k)* classification scenarios. Analysing per-class scores, on average the architecture made the smallest errors when compared with other DNN models.
- 3D CNN [11]—In the reference paper [19] this architecture achieved the best result for two of the three tested datasets. It scored high in our HTC scenario, in particular for the *E(21)* image that was challenging for most of the tested models. In the HIC scenario its results were on par with the rest of architectures. However, it had the highest per-class errors in this scenario among tested models (see Table 5 and Table 6). The training of this architecture was sometimes unstable which resulted in the classifier that assigned all pixels into the one class. Our evaluation procedure described in Section 3.3.2 allowed us to detect and remove these outliers. In the HIC scenario, we observed that the model had significant problems when classifying examples from the *tomato concentrate* and *ketchup* classes. We also observed relatively high standard deviation in the HIC scenario.
- 2D CNN [24]—The results of this network were average among tested models, although in some cases, e.g., *F(2k)→E(7)*, we observed relatively high standard deviation in the HIC scenario.
- 1D CNN [10]—In the reference paper [19] this architecture achieved one of the highest classification accuracies. However, in HIC scenario, it performed worse than other models on average in *F(·)→E(·)* scenarios and also had the highest per-class errors (see Table 6). We also noticed that in some of the HTC scenarios, namely *E(7)* and *E(21)*, its results were also lower than average.
- MLP—Despite its simplicity, MLP achieved competitive results in our experiments, and in some scenarios e.g., HTC *E(7)*, it outperformed other models. This seems consistent with results in [19] or [4]. It suggests that a relatively simple architecture can often compete with more advanced convolutional neural networks.

In order to evaluate the feasibility of combining results from different analysed architectures, we also performed an additional experiment using the ensemble learning approach. This technique achieved slightly better results in the easier HTC scenario but in the more demanding HIC scenario, results of individual architectures were more accurate. The detailed information about this experiment can be found in the Appendix B.

#### 3.5.3. Hyperspectral Blood Stains Classification

Compared to other classes, the *blood* class is relatively easy to classify in *F/frame* images. It can be seen by looking at per-class errors presented in Table 3, Table 4, Table 5 and Table 6. This is consistent with observations in [23]: blood has the unique spectral characteristics in the VIS range, due to the presence of hemoglobin derivatives [17], which makes it distinctive from visually similar substances. Blood is moderately difficult in *E/comparison* images, especially in the HIC scenario. *E/comparison* scenes are darker which introduces noise in the spectra (see [26]). Moreover, spectral responses of hemoglobin change in time [18] which complicates training set creation in the HIC scenario.

The second factor complicating the classification problem is that usually blood stains only partially cover the materials present in the scene. That results in pixels rarely having pure blood spectrum, which is the main reason why *E/comparison* scenes with a complex background structure are more difficult. The usual way to approach this problem is using the spectral unmixing [21] to find blood-related endmembers as e.g., in [57]. However, due to the way unmixing algorithms work, there is no guarantee that the solution they find, e.g., in the form of a linear combination of spectra, will contain spectral components that can be unambiguously identified as blood. In addition, a significant change in the nature of the input data would probably require different hyperparameters and possibly also new architectures of the neural networks used for classification.

Given the importance of result certainty and stability in forensic analysis applications, the problem of blood classification is difficult to solve in a typical machine learning scenario. A way to reduce this difficulty could be to involve the human expert in the classification process e.g., by employing the Active Learning (AL) methods, as e.g., in [58]. There, the classifier can mark examples crucial for training process and request their correct labelling from the expert (oracle). The goal of such a classification is to achieve high accuracy while minimizing the number of queries.

## 4. Conclusions

This paper presents the classification of blood and blood-like substances in hyperspectral images using deep neural networks. Experiments were conducted for a new dataset which has not yet been tested using deep learning models. We performed two series of experiments: the HTC, which is a common scenario in hyperspectral classification, and the HIC, which is less common but useful in terms of applications in forensic science. We tested several architectures from the DeepHyperX library [19], including 1D, 2D and 3D convolutional neural networks, a recurrent neural network and a multilayer perceptron. The presented models were also compared with a Support Vector Machine classifier. In our experiments, we used the output evaluation method based on the analysis of the confusion matrix projection. This evaluation technique allows to detect and remove failed training cases which allows the models to be fully effective and reduces their variance.

Our results show that while the majority of models achieved high accuracy in the HTC scenario, the HIC proved to be more challenging. Interestingly, in the most difficult scenario, where different images were used for training and testing, complex DNN architectures outperformed simpler models like the MLP and the SVM. We also noticed that per-class errors were often the result of mixing physically similar classes such as the *ketchup* and the *tomato concentrate*, while the *blood* class was often classified correctly.

By comparing classification algorithms in the HTC and the HIC scenarios, our study bridges the gap between the two approaches, by examining in detail how different models work in cases of varying difficulty. Along with conclusions from [19], our results help in the assessment of the generalization capability of tested architectures which allows for a more informed choice of a model.

## Figures and Tables

**Table 1:** The results of experiments for the transductive scenario (HTC). In consecutive rows values of Overall Accuracy (OA, %), Average Accuracy (AA, %) and Cohen’s kappa coefficient (κ) are presented for each image. The best OA scores are emphasized.

		SVM	MLP	1D CNN [10]	2D CNN [24]	3D CNN [11]	3D CNN [49]	RNN [14]
	OA:	99.7±0.1	99.7±0.1	99.2±0.3	98.9±0.8	99.4±0.5	99.8±0.2	98.6±3.0
*F(1)*	AA:	99.8±0.1	99.7±0.2	99.5±0.1	99.2±0.7	99.3±0.8	99.9±0.1	97.7±5.5
	κ:	1.00±0.0	1.00±0.0	0.99±0.0	0.98±0.0	0.99±0.0	1.00±0.0	0.98±0.0
	OA:	99.7±0.1	99.6±0.3	99.1±0.2	99.3±0.6	99.8±0.2	99.9±0.1	99.7±0.1
*F(1a)*	AA:	99.7±0.1	99.4±0.5	99.0±0.2	99.4±0.4	99.8±0.4	99.8±0.2	99.7±0.1
	κ:	1.00±0.0	0.99±0.0	0.99±0.0	0.99±0.0	1.00±0.0	1.00±0.0	1.00±0.0
	OA:	99.8±0.1	99.7±0.2	99.3±0.2	99.7±0.1	99.7±0.3	99.8±0.1	99.9±0.1
*F(2)*	AA:	99.7±0.1	99.4±0.7	98.8±0.3	99.4±0.4	99.4±0.5	99.6±0.2	99.8±0.1
	κ:	1.00±0.0	1.00±0.0	0.99±0.0	0.99±0.0	1.00±0.0	1.00±0.0	1.00±0.0
	OA:	99.9±0.1	99.7±0.1	99.2±0.2	98.9±0.5	99.2±1.7	99.8±0.1	96.8±6.2
*F(2k)*	AA:	99.8±0.1	99.5±0.1	98.8±0.3	98.6±0.8	98.7±2.8	99.7±0.2	95.9±7.5
	κ:	1.00±0.0	1.00±0.0	0.99±0.0	0.98±0.0	0.99±0.0	1.00±0.0	0.95±0.1
	OA:	99.8±0.1	99.7±0.1	99.1±0.2	99.4±0.2	99.9±0.1	99.4±1.4	98.9±1.0
*F(7)*	AA:	99.7±0.1	99.5±0.1	98.6±0.2	99.3±0.3	99.9±0.2	98.9±2.7	98.1±2.1
	κ:	1.00±0.0	0.99±0.0	0.99±0.0	0.99±0.0	1.00±0.0	0.99±0.0	0.98±0.0
	OA:	99.8±0.0	99.8±0.0	99.4±0.1	99.6±0.3	99.9±0.1	99.7±0.2	98.7±2.2
*F(21)*	AA:	99.7±0.1	99.6±0.2	99.0±0.2	99.4±0.5	99.8±0.3	99.5±0.3	96.9±5.8
	κ:	1.00±0.0	1.00±0.0	0.99±0.0	0.99±0.0	1.00±0.0	1.00±0.0	0.98±0.0
	OA:	94.5±0.7	93.5±1.7	89.8±0.9	93.4±0.8	86.4±8.2	90.8±4.5	85.1±8.3
*E(1)*	AA:	93.7±0.8	93.1±1.3	89.4±0.6	92.7±1.0	84.8±9.3	89.9±3.7	85.8±6.0
	κ:	0.93±0.0	0.92±0.0	0.87±0.0	0.92±0.0	0.83±0.1	0.89±0.1	0.82±0.1
	OA:	90.6±1.7	90.1±1.9	75.2±1.0	85.5±1.9	80.7±11.3	81.2±9.5	85.3±4.4
*E(7)*	AA:	88.9±1.6	89.1±1.2	74.2±1.0	84.2±2.3	78.2±13.5	79.5±7.7	83.0±6.1
	κ:	0.88±0.0	0.87±0.0	0.69±0.0	0.81±0.0	0.76±0.1	0.76±0.1	0.81±0.1
	OA:	87.8±1.7	89.4±1.6	74.0±2.4	83.3±3.5	94.3±1.8	87.4±3.5	85.0±4.1
*E(21)*	AA:	86.1±1.6	88.1±1.5	72.3±2.3	82.9±2.8	93.3±2.4	86.9±3.4	83.0±3.7
	κ:	0.85±0.0	0.87±0.0	0.67±0.0	0.79±0.0	0.93±0.0	0.84±0.0	0.81±0.1

**Table 2:** The results of experiments for the inductive scenario (HIC). In consecutive rows values of Overall Accuracy (OA, %), Average Accuracy (AA, %) and Cohen’s kappa coefficient (κ) are presented for each image pair. The best OA scores are emphasized.

		SVM	MLP	1D CNN [10]	2D CNN [24]	3D CNN [11]	3D CNN [49]	RNN [14]
	OA:	55.9±2.9	57.8±3.6	46.4±2.3	48.5±5.4	58.1±2.8	53.8±3.2	66.8±3.4
*F(1)*→*E(1)*	AA:	58.6±3.1	60.3±3.3	48.4±2.4	50.6±5.4	55.2±3.6	54.6±2.9	68.2±3.7
	κ:	0.48±0.0	0.50±0.0	0.36±0.0	0.39±0.1	0.49±0.0	0.45±0.0	0.60±0.0
	OA:	60.7±2.6	64.8±2.0	52.1±1.5	59.0±4.9	59.3±4.4	66.6±2.1	71.8±0.9
*F(1a)*→*E(1)*	AA:	62.0±3.1	66.0±1.9	53.1±1.5	60.1±4.7	58.2±4.4	67.6±1.7	71.6±1.2
	κ:	0.53±0.0	0.58±0.0	0.43±0.0	0.51±0.1	0.51±0.1	0.60±0.0	0.66±0.0
	OA:	56.5±5.0	58.7±2.0	49.7±1.0	57.0±2.3	55.3±5.0	63.0±2.9	62.2±1.2
*F(2)*→*E(7)*	AA:	58.7±6.0	61.3±2.2	52.1±0.9	59.1±1.9	53.6±6.9	65.1±3.2	61.7±1.7
	κ:	0.48±0.1	0.50±0.0	0.40±0.0	0.48±0.0	0.45±0.1	0.55±0.0	0.54±0.0
	OA:	52.9±2.6	58.8±1.2	45.7±0.9	51.2±7.1	52.4±7.7	57.3±2.7	59.6±4.7
*F(2k)*→*E(7)*	AA:	54.9±3.1	60.9±1.3	47.1±1.0	52.4±5.8	52.7±6.9	59.0±3.2	57.4±4.5
	κ:	0.43±0.0	0.50±0.0	0.35±0.0	0.41±0.1	0.43±0.1	0.48±0.0	0.51±0.1
	OA:	54.7±2.1	57.2±2.0	47.2±0.8	54.3±2.0	60.3±3.5	59.8±2.6	63.6±3.8
*F(7)*→*E(7)*	AA:	59.4±2.0	60.2±2.2	50.8±0.7	56.1±2.3	57.8±5.0	62.9±2.5	65.3±3.7
	κ:	0.46±0.0	0.48±0.0	0.37±0.0	0.45±0.0	0.52±0.0	0.52±0.0	0.56±0.0
	OA:	45.4±1.7	49.7±2.4	44.0±1.4	49.2±1.6	54.4±3.4	57.2±1.3	56.3±3.1
*F(21)*→*E(21)*	AA:	48.3±2.4	51.9±1.9	45.7±1.7	51.0±1.3	51.4±3.6	59.1±1.4	55.0±2.0
	κ:	0.35±0.0	0.40±0.0	0.33±0.0	0.39±0.0	0.45±0.0	0.49±0.0	0.47±0.0
	OA:	98.2±0.5	97.5±0.6	97.0±0.3	96.9±0.5	80.0±12.1	98.1±0.5	90.4±2.0
*F(2)*→*F(2k)*	AA:	97.7±0.6	96.7±0.8	95.8±0.5	95.6±0.8	77.4±12.1	97.2±0.6	87.2±2.5
	κ:	0.98±0.0	0.97±0.0	0.96±0.0	0.96±0.0	0.75±0.1	0.97±0.0	0.88±0.0
	OA:	99.5±0.2	99.2±0.3	98.8±0.3	96.5±2.1	85.9±10.6	99.2±1.0	93.0±6.0
*F(2k)*→*F(2)*	AA:	99.4±0.3	98.8±0.5	98.5±0.2	96.6±1.9	82.5±13.9	99.2±0.9	93.2±6.2
	κ:	0.99±0.0	0.99±0.0	0.98±0.0	0.96±0.0	0.82±0.1	0.99±0.0	0.91±0.1

**Table 3:** Per-class percentage of errors for each method, aggregated over transductive experiments with *F/frame* images (first part of the HTC experiment).

	SVM	MLP	1D CNN [10]	2D CNN [24]	3D CNN [11]	3D CNN [49]	RNN [14]	(Mean)
B	0.1	0.1	0.3	0.5	0.1	0.1	0.5	0.3
K	0.5	0.7	1.7	1.1	0.4	0.6	1.2	0.9
AB	0.2	0.3	1.2	0.9	0.5	0.2	3.7	1.0
PP	0.1	0.4	0.3	0.4	0.4	0.3	0.4	0.3
TK	0.5	1.1	2.2	1.4	0.6	0.9	5.4	1.7
AP	0.1	0.2	0.6	0.5	0.9	0.5	0.2	0.4
(mean)	0.2	0.5	1.0	0.8	0.5	0.4	1.9	0.8

**Table 4:** Per-class percentage of errors for each method, aggregated over transductive experiments with *E/comparision scene* images (second part of the HTC experiment).

	SVM	MLP	1D CNN [10]	2D CNN [24]	3D CNN [11]	3D CNN [49]	RNN [14]	(Mean)
B	9.7	7.9	12.6	10.5	15.6	9.9	12.0	11.2
K	10.2	8.9	26.0	16.2	17.6	15.8	12.9	15.4
AB	18.3	17.7	43.1	21.4	24.6	22.2	37.5	26.4
PP	1.5	1.2	2.3	2.5	1.8	1.9	2.0	1.9
TK	15.3	15.1	26.4	16.4	18.7	22.0	20.8	19.2
AP	4.8	6.4	10.9	9.4	7.1	11.1	8.1	8.3
(mean)	10.0	9.5	20.2	12.7	14.2	13.8	15.6	13.7

**Table 5:** Per-class percentage of errors for each method, aggregated over inductive experiments with *F/frame* images (second part of the HIC experiment, *F(2)↔F(2k)*).

	SVM	MLP	1D CNN [10]	2D CNN [24]	3D CNN [11]	3D CNN [49]	RNN [14]	(Mean)
B	0.1	0.1	0.1	1.7	10.6	0.0	2.4	2.1
K	1.8	2.4	2.9	4.0	17.0	2.6	8.8	5.6
AB	0.5	0.8	1.7	3.2	16.1	1.3	38.1	8.8
PP	0.9	1.0	1.6	1.9	3.9	0.8	0.3	1.5
TK	4.8	8.1	9.2	11.2	43.4	4.6	8.6	12.8
AP	0.8	1.1	1.7	1.7	32.4	1.5	0.4	5.6
(mean)	1.5	2.2	2.9	3.9	20.6	1.8	9.8	6.1

**Table 6:** Per-class percentage of errors for each method, aggregated over inductive experiments with *F(i)→E(j)* images (first part of the HIC experiment).

	SVM	MLP	1D CNN [10]	2D CNN [24]	3D CNN [11]	3D CNN [49]	RNN [14]	(Mean)
B	35.3	30.4	43.8	39.8	44.8	29.4	42.1	38.0
K	48.4	55.1	84.6	73.8	80.8	72.2	42.7	65.4
AB	83.6	82.2	83.4	81.6	77.3	76.7	85.3	81.4
PP	32.0	29.6	38.8	34.0	5.7	16.9	9.0	23.7
TK	20.2	13.9	16.3	13.3	39.3	13.2	14.7	18.7
AP	36.7	26.0	34.4	27.5	21.1	23.3	20.1	27.0
(mean)	42.7	39.5	50.2	45.0	44.9	38.6	35.6	42.4

## Appendix A. Running Times of Methods

In Table A1 mean running times with standard deviation (in seconds) for each method are presented. These values include not only the training/test time but also data loading time, application of preprocessing, etc. In some cases (*E/comparison scenes*, *F(2)* and *F(2k)*) test times were separated into the HTC and HIC scenario. This is due to the fact that test sets designated for HIC have more pixels than for the HTC scenario. Except for the 2D CNN [24] network, the time of the full test pipeline was slightly greater than 10 s. The bigger differences were noted in the running times of the training part. The fastest calculations were performed by MLP and 3D CNN [49] architectures. A significantly longer computation time has been observed in the case of 2D CNN [24] network. These differences result e.g., from the complexity of architectures and numbers of layers. Furthermore, running times for *E/comparison scenes* are shorter than for *F/frames* because the number of non-background pixels is smaller in the first case.

**Table A1:** The mean computation time with standard deviation (in seconds) of the full pipeline for each method. Consecutive rows present run times for all images, both for the training and the test of the given algorithm.

		SVM	MLP	1D CNN [10]	2D CNN [24]	3D CNN [11]	3D CNN [49]	RNN [14]
*F(1)*	training:	72.2±0.8	27.6±0.5	69.4±1.1	251.7±1.9	44.1±1.6	24.4±0.7	32.7±0.9
	test:	4.2±0.8	10.9±0.3	9.3±0.5	76.4±1.6	11.0±0.0	11.7±0.5	14.1±0.3
*F(1a)*	training:	65.8±0.7	25.3±0.5	65.3±2.7	241.3±0.5	41.0±0.5	24.9±1.1	32.0±1.1
	test:	4.1±0.8	12.2±0.8	10.8±0.4	76.3±2.0	11.0±0.0	11.3±0.5	13.7±0.5
	training:	60.4±0.8	25.4±0.5	63.0±1.3	234.6±0.5	39.7±0.8	23.4±0.5	29.7±0.5
*F(2)*	test (HTC):	4.1±0.8	11.4±1.0	9.6±0.5	76.1±1.6	10.9±0.3	11.3±0.5	14.2±0.6
	test (HIC):	9.4±1.5	12.0±0.0	10.6±0.5	76.9±0.7	10.9±0.3	11.4±0.5	14.1±0.3
	training:	53.1±0.8	26.0±0.5	62.1±1.3	251.0±0.7	44.0±1.8	24.3±0.5	32.1±0.3
*F(2k)*	test (HTC):	4.1±0.9	13.1±0.3	12.2±0.4	98.8±0.6	14.2±0.4	13.8±0.4	16.7±0.5
	test (HIC):	9.6±1.9	13.7±0.7	12.7±0.5	98.9±0.7	13.4±0.5	14.2±0.4	17.3±0.5
*F(7)*	training:	73.5±1.2	27.7±0.5	70.6±1.1	251.3±0.5	43.0±0.5	25.7±0.9	33.1±1.3
	test:	4.6±0.9	11.1±0.6	10.6±1.3	77.3±0.5	11.0±0.5	11.0±0.5	13.7±0.7
*F(21)*	training:	55.6±0.7	24.4±0.5	60.8±0.8	226.0±0.0	40.0±1.8	24.7±0.5	31.8±1.8
	test:	4.2±0.7	11.3±0.5	9.3±0.5	76.7±0.5	10.9±0.3	11.3±0.5	13.8±0.4
	training:	15.3±0.7	12.9±0.3	27.3±0.8	132.6±0.5	18.9±0.3	13.1±0.6	18.9±0.7
*E(1)*	test (HTC):	4.1±0.7	12.0±1.2	10.7±0.5	76.8±0.6	10.8±0.4	11.3±0.5	13.9±0.3
	test (HIC):	8.5±1.6	10.3±0.5	8.9±0.3	74.5±2.8	10.8±0.4	11.1±0.3	13.4±0.5
	training:	7.1±0.6	9.0±0.0	15.6±0.5	101.7±0.5	12.8±0.4	9.0±0.0	14.1±0.9
*E(7)*	test (HTC):	3.9±0.6	11.9±0.6	10.6±0.5	77.0±1.2	10.8±0.4	11.2±0.4	14.1±0.6
	test (HTC):	8.4±1.6	10.3±0.5	9.3±0.7	75.4±1.9	10.8±0.5	11.1±0.6	13.5±0.8
	training:	9.9±0.7	10.0±0.0	20.0±0.0	114.1±0.7	15.1±0.3	10.1±0.3	13.8±0.4
*E(21)*	test (HTC):	4.0±0.6	11.4±0.7	10.9±0.3	73.9±2.2	10.7±0.5	11.1±0.3	13.4±0.5
	test (HIC):	8.7±1.4	10.2±0.4	8.9±0.3	77.1±1.0	10.6±0.5	11.1±0.3	13.7±0.5

## Appendix B. Ensemble Methods

The list of proposed architectures is limited to singular neural networks. Those can be used to build more complicated classification algorithms that can be applied to the problem. The convolutional neural networks have been shown to achieve promising results in committee setting, as noted in [59]. That approach has been successfully explored in GenP classifier in [60] (joint with SVM) or [61] which explicitly explores ensembles of deep learning neural networks for HSI images.

The diversity statistics of the results, measured as the percentage of points in the test set on which the classifiers disagree can be found in Table A2. In the HIC scenario its value can be as high as 0.46. Such a high diversity may suggest that in this particular case, committee approaches might prove useful. However, while high diversity is indication to consider ensemble approaches, it does not translate into higher accuracy of the combined classifier in a straightforward fashion.

Looking at the result of majority voting with random tiebreaker, presented in the Table A2, we can see that in the HTC scenario this result is in most cases better than the result of any individual classifier. However, in a more realistic HIC scenario, there are often multiple networks from a committee that perform better than the community as a whole. This suggests that a more sophisticated ensemble may be needed, e.g., using vote weighing or a modified combiner.

These preliminary results show that while this work focuses on individual neural networks performance, an expansion into multi network classifiers is feasible.

**Table A2:** The results of the ensemble learning experiment, assessing the feasibility of combining outputs of analyzed classifiers. The table presents the minimum, median and maximum diversity for all pairs of classifiers applied to every scenario, accuracy of majority voting and position of this accuracy score when compared to classifiers from Table 1 and Table 2 (1—highest accuracy, 8—lowest accuracy).

Scenario	Min Diversity	Med Diversity	Max Diversity	Majority Voting	Position
*F(1)*	0.0024	0.0099	0.0215	0.9983	1
*F(1a)*	0.0028	0.0065	0.0104	0.998	3
*F(2)*	0.0015	0.0036	0.0085	0.9986	2
*F(2k)*	0.0021	0.032	0.219	0.9984	3
*F(7)*	0.0021	0.0077	0.0154	0.9984	2
*F(21)*	0.0017	0.0046	0.0161	0.9984	2
*E(1)*	0.0617	0.1529	0.276	0.9534	1
*E(7)*	0.0751	0.2079	0.3728	0.9062	1
*E(21)*	0.0951	0.2083	0.3441	0.9077	1
*F(1)*→*E(1)*	0.1413	0.2481	0.4669	0.5739	4
*F(1a)*→*E(1)*	0.1583	0.2501	0.4868	0.6556	3
*F(2)*→*E(7)*	0.1563	0.2829	0.4616	0.6005	3
*F(2k)*→*E(7)*	0.198	0.2988	0.5253	0.5443	4
*F(7)*→*E(7)*	0.1263	0.2684	0.4235	0.5662	5
*F(21)*→*E(21)*	0.1701	0.2981	0.4661	0.5279	4
*F(2)*→*F(2k)*	0.0143	0.0971	0.2783	0.9813	2
*F(2k)*→*F(2)*	0.0084	0.0708	0.3257	0.9943	2
